# Evaluation of Cancer Survivors’ Experience of Using AI-Based Conversational Tools: Qualitative Study

**DOI:** 10.2196/77390

**Published:** 2025-11-14

**Authors:** Saif Khairat, Hanna Mehraby, Safoora Masoumi, Melissa Coffel, Callie Rockey-Bartlett, Andrea Huang, William Wood, Ethan Basch

**Affiliations:** 1 Carolina Health Informatics Program, University of North Carolina at Chapel Hill, 428 Carrington Hall, Campus Box 7460, Chapel Hill, NC, 27514, United States, 1 9198435413; 2 School of Nursing, University of North Carolina at Chapel Hill, Chapel Hill, NC, United States; 3 Lineberger Comprehensive Care Center, University of North Carolina at Chapel Hill, Chapel Hill, NC, United States; 4 Gillings School of Global Public Health, University of North Carolina at Chapel Hill, Chapel Hill, NC, United States

**Keywords:** cancer, survivors, artificial intelligence, user experience, adoption, facilitators, barriers

## Abstract

**Background:**

Cancer survivorship is a complicated, chronic, and long-lasting experience, causing uncertainty and a wide range of physical and emotional health concerns. Due to the complexity of cancer, patients often seek out multiple sources of health information to better understand the aspects of their cancer diagnosis. The high variability among patients with cancer presents significant challenges in treatment, prognosis, and overall disease management. Artificial intelligence (AI) chatbots can further personalize cancer care delivery. However, there is a knowledge gap regarding cancer survivors’ perceived facilitators and barriers to adopting and using AI chatbots.

**Objective:**

In this study, we examined cancer survivors’ experiences of using existing AI chatbots and identified their facilitators and barriers to the adoption of AI chatbots.

**Methods:**

We conducted a qualitative study to investigate the perceptions of cancer survivors, conducting semistructured interviews to understand their prior use of existing AI chatbots in general. We asked the participants about their perceptions regarding AI chatbot acceptability and comfort level; trust and adherence; and concerns, barriers, and suggestions. We used the Consolidated Criteria for Reporting Qualitative Research (COREQ) checklist for this qualitative report.

**Results:**

Of 21 participants, 17 (81%) were female patients with breast cancer, 15 (71%) were aged 50 to 64 years, 19 (90%) were White, and 9 (43%) had a graduate degree. Participants’ responses were grouped into three overarching themes: (1) patients’ perceptions of interacting with chatbots compared to health care professionals, (2) patient-chatbot interaction, and (3) chatbot information processing. All participants who were interviewed reported that they would prefer interacting with health care professionals over a chatbot. The lack of empathy shown by chatbots was a major concern among cancer survivors. Many patients criticized chatbots for tending to provide a general overarching response to their questions rather than being specific to their cancer diagnosis. The main concerns of cancer survivors with using chatbots were the overabundance of general information that was often not relevant to their diagnosis and privacy of patient information.

**Conclusions:**

The findings of this study underscore the critical importance of empathetic responses during AI chatbot interactions for cancer survivors, as the lack of personalized and emotional responses can lead to distrust and frustration. Clinically, these tools should be integrated as supplementary resources to enhance patient engagement while preserving essential human support. Policymakers need to develop guidelines that promote responsible use of AI in cancer care, prioritizing patient confidentiality and trustworthiness. AI chatbots have the potential to significantly improve the support provided to cancer survivors, but it is crucial to address the identified barriers and enhance user acceptance.

## Introduction

Cancer is a complex and highly individualized disease [1]. Cancer survivorship, which extends from the time of diagnosis through the balance of life, is a complicated, chronic, and long-term experience that can cause uncertainty and a wide range of physical and emotional health concerns [2]. Due to the complexity of cancer, patients often seek out multiple sources of health information to better understand the aspects of their cancer diagnosis. Patients with cancer show extreme variability in disease progression, treatment response, and side effects, at least partly due to genetic differences, tumor heterogeneity, and immune system variations [3,4]. This variability makes treatment standardization and outcome prediction difficult, necessitating personalized treatment strategies that general medical guidelines may struggle to accommodate accurately [5-7]. Patients with cancer also require continuous monitoring and tailored supportive care to address complicated prognoses, as patients with similar diagnoses may experience vastly different disease trajectories and treatment-related side effects [8]. These complexities underscore the need for the provision of personalized, evidence-based oncological information tailored to unique cancer cases to optimize treatment efficacy and improve patient outcomes [9].

Artificial intelligence (AI) chatbots have revolutionized health information seeking [10]. These large language model (LLM)–based conversational agents enhance health care by improving access to medical information, supporting clinical decision-making, and automating administrative tasks, reducing the burden on health care professionals (HCPs) [11,12]. They also improve patient engagement through symptom tracking, medication reminders, and mental health support while aiding medical education with up-to-date resources [13]. Additionally, LLMs contribute to early disease detection by analyzing patient data trends, ultimately improving diagnosis and treatment outcomes.

Previous research evaluating chatbots predominantly focuses on accuracy and does not sufficiently examine user acceptance [10,14]. One study reported that chatbots may aid in drafting responses to patient questions, although there were concerns about the level of empathy provided compared to humans [15,16]. Chatbots may be able to provide personalized responses—as a supplement to medical care—about treatment side effects, pain management options, receiving follow-up care, and navigating complex health information. Current research evaluating responses to cancer-related prompts using generative pretrained transformer (GPT)–based chatbots indicates that there is a need for further development and optimization for health care implementation [11-15]. Recent developments in cancer-focused chatbots such as OncoGPT (OncoAI Labs), Prostate Cancer Info (Prostatica Digital Health), IBM watsonx Assistant, and other AI-guided bots underscore the growing interest and potential for chatbots in oncology [16-19]. However, there is a knowledge gap regarding the barriers and facilitators that influence the adoption and use of AI chatbots among cancer survivors [20].

Examining the user acceptance of chatbots in cancer care is essential for determining how patients, caregivers, and HCPs incorporate these tools into their decision-making and support systems [21]. Patients with cancer require highly personalized and trustworthy information. If they do not trust chatbots or find chatbots useful, these tools may fail to provide meaningful support. Additionally, understanding user acceptance helps to identify barriers, such as privacy concerns, lack of emotional support, and data risks, which can inform the design of more patient-centered and clinically reliable chatbot interventions. High user acceptance can enhance patient education, symptom management, and emotional well-being, ultimately improving health outcomes [22]. Without proper acceptance, even the most advanced chatbot technology may remain underused, limiting its potential impact in oncology care. This study aimed to understand the user experience of cancer survivors using AI chatbots and characterize the facilitators and barriers to the adoption of AI chatbots.

## Methods

### Study Design

This qualitative study used a descriptive phenomenological design to explore cancer survivors’ experiences with AI chatbots. Phenomenology was chosen to capture the participants’ lived experiences and perceptions [23]. Our research team used the Consolidated Criteria for Reporting Qualitative Research (COREQ) checklist (Checklist 1) for the topics that were applicable to this qualitative report [24].

### Participants

Our study included adults older than 18 years who met the definition of breast and prostate cancer survivors and who received cancer care in the United States. We chose to focus on breast and prostate cancer, as these are the most common cancer types in the United States and have the highest survival rates [25]. Experience of using a chatbot was not required. To determine the eligibility of cancer survivors for our study, we used the definition provided by the National Cancer Institute [26]: “An individual is considered a cancer survivor from the time of diagnosis through the balance of life.” Treatment status was not considered an inclusion or exclusion criterion. By scanning a QR code in the study flyer, interested individuals were able to access a Qualtrics screening survey, which was used to determine the eligibility of cancer survivors (Multimedia Appendix 1). The email addresses of interested individuals were collected through the Qualtrics survey, and eligible cancer survivors were invited by email to participate in our study. No eligible participants refused participation or dropped out.

### Ethical Considerations

The study was determined to be exempt from review by the University of North Carolina Institutional Review Board (approval number 23‐1451). We recruited participants by sharing an electronic study flyer with local and national cancer survivor organizations. Recruitment was conducted between September 2023 and December 2024. All participants completed a screening survey to determine study eligibility. The screening survey data were anonymized but not deidentified. Informed consent was obtained from all participants. The participants were informed about the ability to opt out of the study at any time. All participants received a US $25 gift card as compensation.

### Recruitment Procedure

Only 4 prostate cancer survivors were recruited. However, this study did not aim to examine the differences in experiences between breast and prostate cancer survivors. Instead, it aimed to include diverse perspectives. We determined that there were similarities in the responses to interview questions between prostate and breast cancer survivors; therefore, we believe that saturation was adequately achieved for both groups.

### Data Collection

The participants were asked to participate in a semistructured interview to share their experiences and perceptions regarding AI chatbots in health care. The interview guide contained an introduction, instructions, and 8 questions separated by section (Multimedia Appendix 1). In total, 4 questions were related to acceptability and comfort level; 2 questions were related to trust and adherence; and 2 questions were related to concerns, barriers, and suggestions. The participants were asked about their experiences with the use of or perceptions about any chatbots, rather than one specific chatbot, to expand the scope of responses. This study was qualitative in nature; therefore, we did not develop a hypothesis for our interview questions.

We used one-time, one-on-one Zoom interview sessions (version 6.5.12; Zoom Communications Inc) as our setting for data collection, with only the participant and researchers present. One of 3 research assistants conducted the interviews in this study (HM, SM, and MC). Two research assistants (HM and SM) were trained by a third research assistant (MC) while they shadowed her during the initial interviews, to ensure consistency in interview technique. The interviews were 20 to 30 minutes long and were conducted between September 10, 2024, and December 23, 2024. No repeat interviews were conducted. Field notes were collected during each interview. Participants were recruited until thematic saturation was reached for both cancer groups combined. We defined thematic saturation as the point at which no new codes or themes emerged, as described by Guest et al [27]. We determined that saturation had been achieved once responses to interview questions became repetitive across prostate and breast cancer survivor participants, and no substantial differences in themes were observed. All interviews were audio recorded on a secure, password-protected recording device. Audio recordings were uploaded to a secure, password-protected, Health Insurance Portability and Accountability Act–compliant server, along with audio file transcripts and field notes taken during interviews to capture key points. Before the interview, the participants were assigned a number to eliminate the possibility of identifying them from their responses. Audio recordings were separated by participant number, and no transcripts were returned to the participants after the interviews were concluded.

### Data Analysis

An inductive thematic analysis was used to identify themes and patterns directly from the data collected in the interviews [23]. This method helped us to gain a nuanced understanding of the data while allowing for the emergence of unexpected themes and patterns common across participant responses. A codebook was developed to facilitate the analysis of patient transcripts via a priori coding based on the interview guide and emergent coding during the interview process. One researcher (MC) initially developed a draft of the codebook, and it was then refined by 4 researchers (HM, CR-B, AH, and SM). The overarching codes covered interactions with AI, perceptions of AI, perceptions of HCP communication, and cancer-specific patient experiences. Subcodes were then created within these parent codes to include specific patient concerns. Each interview was independently coded, by random assignment, by 2 of 5 authors (HM, CR-B, AH, SM, and MC). Interview transcripts were coded in Dedoose (version 9.2.22; SocioCultural Research Consultants, LLC). Coding discrepancies were discussed and resolved through consensus between 5 authors (HM, CR-B, AH, SM, MC, and SK). Data analysis was conducted after the conclusion of participant recruitment and the interview process. Stakeholders and participants were not involved in the development of codes or interpretation of findings.

### Overarching Themes

After analyzing the coded transcripts in Dedoose, we identified various patterns that intersected across participant interviews. Three overarching themes emerged from the patterns identified. After coming to an agreement on each theme, 3 authors (HM, AH, and CR-B) retrieved all text with codes related to the respective themes. For each theme, relevant participant quotations were pooled into one table. Minor themes, or subthemes, corresponding to each theme were identified. Subthemes represented experiences or perceptions shared by multiple participants and contributed to a broader theme. Afterward, we independently ranked the relevance of each excerpt based on how adequately it illustrated each respective theme. We selected 2 excerpts for each subtheme. Each quotation was identified by participant number. Findings from these excerpts were then identified as either a facilitator or a barrier to the use of chatbots for the relevant themes.

## Results

### Principal Findings

Of 21 participants, 17 (81%) were female patients with breast cancer, 15 (71%) were aged 50 to 64 years, 19 (90%) were White, and 9 (43%) had a graduate degree (Table 1).

**Figure 1.**
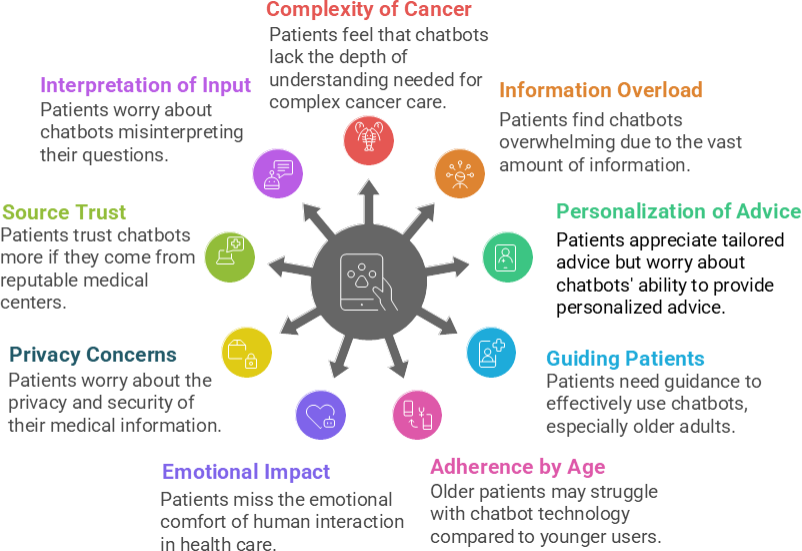
Participants’ experiences with artificial intelligence chatbots.

The experiences of patients with cancer interacting with a chatbot while receiving cancer care centered mainly around three themes: (1) patients’ perceptions of interacting with chatbots compared to HCPs, (2) patient-chatbot interaction, and (3) chatbot information processing. Theme 1 describes the factors influencing patients’ perceptions and experiences of interacting with and using chatbots versus interacting with HCPs. Theme 2 describes how patients can use chatbots, how chatbots present information, and how patients interpret chatbot responses. Theme 3 describes patient perceptions regarding how chatbots process information to create responses. Under each theme, we identified 3 to 4 subthemes. Barriers and facilitators were identified for themes 2 and 3. Barriers and facilitators were not applicable to theme 1, as the findings focused on patients’ general perceptions of chatbots relative to HCPs rather than on specific interactions with a chatbot where barriers or facilitators would emerge. Furthermore, because all participants indicated that they would prefer to seek medical advice about their cancer from an HCP rather than a chatbot, no barriers or facilitators were identified for theme 1, as these were merely factors that influenced patient preference for HCPs. Figure 1 summarizes all findings.

**Table 1.** Demographics of participants.

Characteristic	Prostate cancer survivors (n=4), n (%)	Breast cancer survivors (n=17), n (%)	Total participants (N=21), n (%)
Age group			
18‐34 years	0 (0)	0 (0)	0 (0)
35‐49 years	0 (0)	5 (29)	5 (24)
50‐64 years	3 (75)	12 (71)	15 (71)
65‐79 years	1 (25)	0 (0)	1 (5)
Sex			
Male	4 (100)	0 (0)	4 (19)
Female	—^a^	17 (100)	17 (81)
Race/ethnicity			
Black or African American	0 (0)	1 (6)	1 (5)
White	4 (100)	15 (88)	19 (90)
Hispanic or Latino	0 (0)	1 (6)	1 (5)
Education			
Associate degree	0 (0)	2 (12)	2 (10)
Bachelor degree	2 (50)	8 (47)	10 (48)
Graduate degree	2 (50)	7 (41)	9 (43)
Employment status			
Unemployed	2 (50)	5 (29)	7 (33)
Part-time	1 (25)	1 (6)	2 (10)
Full-time	1 (25)	11 (65)	12 (57)

a Not applicable.

### Theme 1: Patient Perceptions of Chatbots Compared to HCPs

There were ultimately two factors that influenced patients’ preference for an HCP over a chatbot: (1) complexity of cancer and (2) information overload (Table 2). All participants indicated that they would prefer to seek medical advice for their cancer from an HCP rather than a chatbot.

While all participants indicated that they would prefer HCP input, some participants shared instances or characteristics of chatbots that would increase their likelihood of using a chatbot before seeking information from an HCP. One participant stated that the risk that is posed to their health and cancer outcomes affects whether or not they trust a chatbot’s response. The participants generally reflected that they do not feel the need to consult with an HCP if their question is about a minor issue, such as a minor side effect of a procedure. Furthermore, the participants shared that if the chatbot provided evidence-based sources to consult along with its response, they would be less likely to follow up with their HCPs.

**Table 2.** Perceptions of patients with cancer regarding interactions with chatbots, categorized by subtheme.

Subtheme	Illustrative quote	Patient characteristics			
		Cancer type	Age group (years)	Race/ ethnicity	Education
Complexity of cancer	“I think it’s because I feel that [HCPs] know more specifically of what’s going on with me personally and they’re able to see the big picture where I’m just not sure I can go in there and give the chatbot more details, but I still don’t know if they don’t know my full medical history and record. It’s always good to just double-check and verify with my physician or oncologist just to make sure.”	Breast cancer	35‐49	White	Graduate degree
Complexity of cancer	“If it was something more specific like eat more ginger, I don’t know, just as an example that maybe I need to check with my doctor to confirm that that’s not going to interact with any of my medications that I’m already on or something like that because the chatbot is not going to know all of my medical history, all of my medications, that sort of thing. If it’s more of a general question then I might not check with my doctor.”	Breast cancer	35‐49	White	Bachelor’s degree
Information overload	“[AI] takes the personal care, the customer care, customer service out of medicine, if you will. That’s how we were brought up, you consult with a physician, they’re supposed to know your entire history. [Physicians] are supposed to be able to pull from parts A, B, and C into your situation and know that this could happen to you or this has happened to you as opposed to an AI is just going to answer one direct thing. And I don’t know how far it would go down a rabbit hole, and I don’t know if they’re designed to go down rabbit holes, but physicians are, and they know when to stop and when not to stop.”	Breast cancer	50‐64	White	Bachelor’s degree
Information overload	“There are so many different options available that I found...which led me to spending an inordinate amount of money and time looking into certain things. When I had the wonderful opportunity to talk to one of our professors at UNC surgeon, it was probably one of the shortest conversations I ever had because...I said, I have these questions, and she just plain told me, here’s the way to go, and I felt comfortable with that.”	Prostate cancer	50‐64	White	Graduate degree

#### Complexity of Cancer

The complexity of individual cancer cases was a major reason that patients preferred to seek advice from an HCP. Participants shared that empathy from HCPs is crucial for patients during their cancer journey, especially if they are dealing with a complex and difficult diagnosis. One participant reflected on how they dealt with more emotional and physical issues during their second diagnosis, causing them to consult with physicians on whether their experience was normal. Their physicians were able to empathize with their fear and encourage them that their treatment path was appropriate for their diagnosis. The participants shared that they felt that the chatbot lacked the level of empathy they would have experienced while speaking to a human.

Given the potential impact of lifestyle on cancer outcomes, patients preferred communication about changing behaviors to address emerging health issues and concerns with HCPs. Participants reflected that they felt the need to seek additional answers for cancer more than they would for other health issues. Because the grade or stage of cancer can quickly change, receiving advice from HCPs for steps to manage cancer and associated symptoms is more critical. Additionally, the participants voiced that HCPs know the more specific big picture details about individual cases than a chatbot would. They expressed that, because chatbots do not have access to a patient’s full medical history, they would feel more confident in following a chatbot’s advice after they verify the information with their physicians or oncologists.

Several participants described instances where they would consult with an HCP for more input if they had a condition or were making a lifestyle change, especially if it could affect their cancer management and outcomes. The participants shared that if the chatbot recommended that a patient make a lifestyle change, they would feel the need to consult with their physician to confirm that it would not interact with their current treatment plan. Seeking HCP input on recommendations from a chatbot was especially important to the participants because chatbots do not know a patient’s medical history.

#### Information Overload

Participants reported that chatbots tend to provide too much information in response to questions, causing uncertainty about which recommendations to follow. One participant shared that the chatbot provided them with an overwhelming amount of information, causing them to spend a large amount of time and money trying to navigate which advice to follow. However, once they sought advice from an HCP, the HCP immediately gave them directions on what to do next, which the participant trusted.

Furthermore, HCPs have knowledge about the nuances of cancer cases and can analyze information that could affect well-being on a certain treatment plan, including individual medical history, medications, and lifestyle behaviors. Additionally, they know the appropriate amount and type of information to be provided to patients to ensure that they understand their case and the proper treatment plan correctly. The participants reflected that chatbots do not have the capacity to do this because they do not tend to take patient-centered approaches when disseminating information.

### Theme 2: Patient-Chatbot Interaction

Theme 2 included four subthemes: (1) personalization of advice, (2) emotional impact of AI chatbot interactions, (3) guidance for patients regarding the effective use of AI chatbots, and (4) adherence to AI chatbot advice by age group (Table 3). Of the top 8 quotes identified under this theme, 4 represented facilitators, and 4 represented barriers.

**Table 3.** Perceived barriers and facilitators of patient-chatbot interaction among patients with cancer, categorized by subtheme.

Subtheme	Illustrative quote	Patient characteristics				Barrier or facilitator
		Cancer type	Age group (years)	Race/ ethnicity	Education	
Personalization of advice	“If [chatbots] could understand the treatment plans, the pathways, and future follow-up care...because it’s different for every type of breast cancer...if they could better understand what the particular person’s diagnosis is, then that would help them give a better, more focused, targeted answer.**”**	Breast cancer	35‐49	White	Graduate degree	Facilitator
Personalization of advice	“You maybe don’t have to re-ask questions, don’t have to revisit topics. For example, I was having trouble with urinary incontinence. The chatbot would already have our previous conversations and then could go deeper into the subject or skip some of the early information gathering.”	Prostate cancer	50‐64	White	Bachelor’s degree	Facilitator
Guidance for patients regarding the effective use of AI^a^ chatbots	“If there were vetted sources, in particular AI engines that had been investigated by your doctor’s office and there was a brochure...particularly for older people...that could kind of show you how to use [chatbots] in a safe way and get the right of information, I think that that might work.”	Breast cancer	35‐49	White	Bachelor’s degree	Facilitator
Guidance for patients regarding the effective use of AI chatbots	“...keeping the responses succinct, short so that they were easy for people to understand.... I work in training, and we usually say to write to a sixth-grade level...it would be nice to have [responses] as simple as possible.”	Breast cancer	50‐64	White	Graduate degree	Facilitator
Adherence to AI chatbot advice by age group	“The older population...will have more issues because it wasn’t around back in the day. I came from the generation of a rotary phone dial connected to a cord connected to the wall. There were no cell phones, so I don’t have it. I’m in that kind of middle generation, but I bet you if you talk to older people, they will maybe not understand bots more than me.”	Breast cancer	50‐64	White	Bachelor’s degree	Barrier
Adherence to AI chatbot advice by age group	“The younger generation are going to know [how to use chatbots], but us older people, if you’ve been out of the workforce for a few years and you’re not up to the new electronic things, it’s going to leave some of us behind.”	Breast cancer	50‐64	White	Associate degree	Barrier
Emotional impact of AI chatbot interactions	“[AI] would be a cold option compared with talking to a real person...if you’ve ever experienced anxiety or depression when you’re going through cancer...then you realize that getting answers off the web...is not very comforting. If someone can look you in the eye or you hear their voice on the phone...it’s more comforting.”	Breast cancer	50‐64	White	Graduate degree	Barrier
Emotional impact of AI chatbot interactions	“But a lot of issues that we deal with are anxiety, depression, suicidal tendencies and concern about reaching out for healthcare based on past experiences.... There’s a whole ’nother realm that AI doesn’t give you...and it’s not as personal. I would choose personal every single time.”	Breast cancer	50‐64	White	Bachelor’s degree	Barrier

a AI: artificial intelligence.

#### Facilitators of Patient-Chatbot Interaction

Participants voiced that a key facilitator was AI chatbots providing personalized responses based on a patient’s specific needs. They shared that they would want chatbots to understand their situation and provide a more tailored response to their diagnosis as well as give a focused, targeted answer. For example, the participants said that the benefit of AI chatbots saving previous conversations would be that the participants would not have to repeat questions. Instead, the chatbot could skip the phase of gathering information that was already discussed and instead go deeper into the subject to facilitate personalization of advice. Additionally, the participants said it would be helpful if chatbot responses were short and easy to understand so that the information would not be overwhelming. Ultimately, understanding cancer is complex, and it would be better if chatbots kept the answers as simple as possible. One participant voiced that, because AI is going to be the future, it would be helpful if there were AI tools already reviewed and approved by their HCP and if educational resources were provided at clinics, such as a brochure to guide patients on how to effectively use AI chatbots. They emphasized that this is especially important for increasing trust among older generations.

#### Barriers to Patient-Chatbot Interaction

Adherence to AI chatbot advice varied by age group. The older the participants, the less likely they were to follow chatbot advice. The novelty of chatbots was emphasized to be an influential factor impacting mistrust in chatbot responses among older populations in particular. Participants reflected that the inability of AI chatbots to collect individualized information from patients contributed to the lack of personalized responses. They indicated that they had greater trust in chatbot responses to general questions. However, the participants indicated that they need more detailed responses at times and that chatbots often do not have all the information to give an adequate response because the specific details pertaining to a diagnosis are different for every cancer survivor.

Regarding the emotional impact of AI chatbot interactions, another concern of participants was the lack of empathy in chatbot responses. The participants expressed that cancer is an emotional experience, and many people going through treatment also face mental health challenges such as anxiety, depression, and suicidal thoughts. They described that getting answers from the internet was a colder option compared to speaking with an HCP. It was implied that chatbots only focus on the physical symptoms and there are personal factors that AI chatbots do not address.

### Theme 3: Chatbot Information Processing

Our team identified theme 3 as chatbot information processing. The participants reflected on three subthemes: (1) privacy of information, (2) sources of information, and (3) the chatbot’s interpretation of inputted information. Facilitators and barriers were identified for all subthemes (Table 4).

**Table 4.** Perceived barriers and facilitators of chatbot information processing among patients with cancer, categorized by subtheme.

Subtheme	Illustrative quote	Patient Characteristics				Barrier or facilitator
		Cancer type	Age group (years)	Race/ ethnicity	Education	
Privacy of information	“I guess [my top concern] would be the privacy part. Just making sure that whatever is shared through that tool is kept private and not too many hands or [*sic*] dipping in and information get [*sic*] exposed or taken away. But [privacy] would be my top concern.”	Breast cancer	50‐64	African American/ Black	Associate degree	Facilitator
Privacy of information	“Is there a chance it’s using my information or my questions and feeding that information to insurance companies or things like that?”	Prostate cancer	50‐64	White	Graduate degree	Barrier
Sources used by the chatbot	“I think [using AI chatbots] would depend on the source of the chatbot. I would be more likely to trust one that came from a medical center.”	Breast cancer	50‐64	White	Graduate degree	Facilitator
Sources used by the chatbot	“[The chatbot] may give wrong information. So that’s certainly possible. I wouldn’t trust my life with it for sure.”	Prostate cancer	50‐64	White	Graduate degree	Barrier
Chatbot’s interpretation of input information	“It could be missing context because a bot is going to go based on a knowledge base of information, understanding the question that I’m asking and the most appropriate answer based on [my] questions, but I may not know the right question...”	Breast cancer	50‐64	White	Bachelor’s degree	Barrier
Chatbot’s interpretation of input information	“I think it depends on the language that the person enters into the chatbot.... [The patient] could receive information that could influence their healthcare decisions, that could give them poor outcomes.”	Breast cancer	35-49	White	Bachelor’s degree	Barrier

#### Facilitators of Chatbot Information Processing

Regarding privacy, a participant shared that they would be more comfortable if an explicit button was provided within the interface to ensure that any information provided by the patient to the chatbot would not be collected. In terms of facilitators of trust in the information provided by chatbots, the participants mentioned that information obtained from reliable sources would be more accurate than the information available on the internet. Participants expressed greater trust in sources that were directly linked to medical centers and research. Additionally, the participants shared that if the chatbots provided links to their sources of information, it would allow them to dig deeper and feel more confident about chatbot responses. Additionally, a participant suggested that it would be helpful if chatbots could incorporate and interact with the resources provided to the patient by their HCP.

#### Barriers to Chatbot Information Processing

Privacy was cited as a barrier to engaging with chatbots for health-related questions. Some patients stated that they had not asked cancer- or health-related questions to chatbots because they did not have expectations of privacy. Others were concerned that whatever they shared could be exposed. For example, one participant expressed concerns about chatbots providing their information to insurance companies, which could potentially affect their care. This common concern that patient information would be collected by the chatbots and potentially distributed or accessed by other entities appeared to be a limiting factor for chatbot engagement.

The potential impact on patient decisions and downstream health outcomes was cited as a consideration for trusting chatbot responses. Many participants emphasized the importance of accurate information in their health decisions. The information shared with patients, especially for issues related to cancer, could be a matter of life and death. Participants shared that a chatbot’s responses could impact whether a person seeks care immediately. The influence of chatbot responses in health care decisions and potential poor outcomes was also a shared concern among participants.

Many barriers to trusting chatbot responses were related to where chatbots source their information. Some participants were concerned that chatbots would not be able to distinguish misinformation from information as the source. One participant also felt that, in addition to dispensing potential misinformation, chatbots give out too much information without any filter. A few participants expressed their wariness from experiences of receiving misinformation from AI in general. One participant shared that in their experience, AI provides answers that appear to be real but then provides fabricated author and journal names. Some participants acknowledged that even if chatbots provide sources, users may have differences in their ability to evaluate the quality of the sources. Another participant mentioned that if they had not personally provided the source that they wanted summarized, they would not trust a chatbot’s ability to choose the best sources of information. Together, the potential for chatbots to provide misinformation and subsequently influence health outcomes was a common barrier identified by the participants.

Another barrier identified by the participants was that chatbots may interpret patient questions incorrectly on the basis of the wording used, affecting the way the chatbots source the information. One patient pointed out that they might not know how to word their question to a chatbot to express their concerns and that the chatbot could be missing the context needed to understand their question correctly. Similarly, another patient expressed that the responses and information received from a chatbot are dependent on the language that the person enters into the chatbot. Thus, it appears that some limitations also stem from the limitations of the information and prompts provided by patients.

## Discussion

### Principal Findings

This qualitative study aimed to investigate the user experience of cancer survivors using AI chatbots to seek health information. The participants, who were breast and prostate cancer survivors, evaluated the quality and accuracy of health care advice provided by AI chatbots for breast and prostate cancer. Our findings show that the user experience of cancer survivors centered around their preferences between chatbots and HCPs, perceptions around patient-chatbot interaction, and the information processing ability of chatbots.

We found that the lack of empathy shown by chatbots was a major concern among cancer survivors. Some participants expressed concerns with the lack of empathy in chatbot responses, which explains why they preferred to seek health care advice from HCPs instead of chatbots. HCPs can recognize and respond to patients’ emotional needs. In contrast, a chatbot was seen as more of a cold option, especially for patients who dealt with mental health challenges during their cancer journey. However, this preference also depended on whether a patient expected empathy when seeking health care advice, as multiple participants expressed not wanting empathetic responses from a chatbot. Previous research on patients with cancer suggests a positive association between physician empathy and patient outcomes such as higher satisfaction, decreased emotional distress, and decreased anxiety [28-31]. This emphasis on clinician empathy suggests that the expectation and perception of empathy is a current gap in the adoption of AI chatbots.

The topic of personalization overlapped across all 3 themes. The level of personalization often impacted patient satisfaction with chatbots in response to inquiries. Many patients criticized the general responses provided by chatbots to their questions and would have preferred responses specific to their cancer diagnosis. Participants voiced their preference for chatbots to better understand their specific health condition and to provide a more tailored response. HCPs can provide more tailored responses because of their access to patients’ electronic health records (EHRs), their relationship and communication with their patients, and their knowledge of their patients’ health needs and preferences [32,33]. Chatbots should have access to patients’ EHRs to provide a personalized experience that meets the expectations of cancer survivors.

We found that a primary concern of cancer survivors regarding chatbot use was the overabundance of general information that is often not relevant to their diagnosis. Previous studies report that adults feel overwhelmed by the amount of information on cancer, a phenomenon referred to as cancer information overload [34]. Recent research on the use of AI in urological cancer shows that applying machine learning algorithms is promising for overcoming this information overload, as they enable a more focused and efficient search [35]. Our findings confirm that survivors prefer chatbots that provide tailored information relevant to their health condition without extraneous and irrelevant information.

Privacy and trust were major roadblocks to the adoption of chatbots. Many participants shared their concerns about providing personal information to chatbots because of privacy concerns. “AI explainability” is a term that indicates transparency around the design of chatbots, data storage, security, and the information sources used [36]. The participants voiced concerns around not understanding what happens to information that is inputted into chatbots. Generational differences related to digital literacy may further impact trust and knowledge about how to best use chatbots. Nevertheless, having a chatbot that better details its methods to protect medical data will be important to enhance trust among older groups. The existing literature shows that digital health technologies have the potential to reduce cancer care health disparities and that limited digital health literacy is a barrier to digital technology engagement [36]. Therefore, implementing explainable AI chatbots in the context of cancer care is important to address this variability in digital literacy.

The participants saw merit in chatbots being introduced and recommended by HCPs, as this may enhance trust and self-efficacy. The participants’ feedback suggests that chatbots should not replace their HCPs. Instead, they should be used to supplement HCP interactions. Integrating HCP-endorsed chatbots into care pathways may strengthen patients’ trust and willingness to engage with AI tools. If HCPs can guide patients regarding the safe use of chatbots, patients will feel more confident to use chatbots, especially older individuals who have less trust. Patients may be more willing to trust chatbots if HCPs recommend their use. Including recommended links to vetted medical sources within responses may also reduce the sense of being overwhelmed that patients feel with the overload of information provided by chatbots. It would also direct patients to chatbots that provide informed responses. An HCP-endorsed chatbot, where HCPs seek answers from a chatbot and validate the information before sending it to patients, could enhance trust and improve the provision of tailored responses to patient concerns. However, this could also potentially increase HCP burden and patient wait times for responses. Enhancing the quality of chatbots for use among cancer survivors could directly benefit HCPs and patients by reducing HCP burden and improving the speed at which patient concerns are addressed. Chatbots could provide 24-7 access to information for patients who seek answers to their questions, thereby reducing the volume of patient inquiries that HCPs need to respond to. Additionally, chatbots could deliver immediate responses to patients at any time, reducing wait times and therefore enhancing patient satisfaction and quality of care.

Our findings align with recent systematic reviews of patient perspectives regarding AI in oncology care. For example, recent studies reported that patients prioritize empathy and personalization, consistent with our themes [37,38]. Similarly, another study highlighted that trust and transparency are key determinants of chatbot adoption [39].

### Limitations

There are several limitations to this study. Most participants included in the study identified as White, limiting the generalizability of our findings to other racial and ethnic groups. The study included 4 prostate cancer survivors, not to compare them with breast cancer survivors but to gather diverse perspectives. Similarities in responses indicated that saturation was sufficiently met for both groups. Experiences of different minoritized patient populations may provide insight into their interactions with chatbots in their cancer care journey. Furthermore, all participants had received some type of college education, which may limit the generalizability of these findings to individuals with different educational backgrounds. Education could potentially influence the perspectives around the adoption and use of AI chatbots. Additionally, most patients interviewed in this study were female breast cancer survivors. Perspectives from patients with different cancer diagnoses may provide broader insights into chatbot use in cancer care. Additionally, despite 4 prostate cancer survivors participating in this study, our findings may not be generalizable to all prostate cancer survivors. While the specific findings of this study may not be generalizable to patient populations beyond breast and prostate cancer survivors, they provide valuable insights into patient perceptions of AI chatbots. In addition, the findings are based on participants’ perceptions of self-reported encounters and hypothetical scenarios rather than recorded chatbot use data, which poses some limitations on the ecological validity. Future studies could investigate real-time chatbot interactions to better capture the impact on engagement. Furthermore, while the participants exhibited a range of familiarity with and experience of chatbots, the participants’ digital literacy was not formally assessed. This poses some limitations to the representativeness and generalizability of our study.

### Conclusion

In this study, cancer survivors generally had a positive outlook on the implementation of chatbots in cancer care. Participants’ perceptions of chatbots compared to HCPs suggest that chatbots are more suitable as a supplemental resource than a complete replacement for HCP interactions. The barriers identified by patients provide valuable insight into how chatbots can be improved and tailored to meet the needs of patients with cancer. Transparency in data privacy, reducing information overload, and providing links to trusted sources were commonly shared as potential recommendations to improve chatbots for use in cancer care. Moreover, patient education in the clinical setting on how to use these tailored chatbots and ask the right questions can improve the user experience and patients’ trust in chatbots. The study participants’ perceptions of virtual care and AI chatbots suggest that, while there are perceived benefits of virtual chatbot-based health care, certain aspects of the interface and interaction could potentially be improved to better suit patient needs. Future research could build on the findings of this study by evaluating these identified factors through observations of real-time chatbot interactions, reviews of use patterns, and questionnaires on patients’ perceptions. Integrating a more formal evaluation of digital literacy could also provide greater insight into how chatbots can better suit the needs of patients with a range of technological experience. Future research could also investigate the impacts of different levels of integration of chatbots in medical care. AI chatbots can potentially improve the delivery of valuable health care information for patients with cancer; however, additional work is needed to tailor these chatbots to the expectations and needs of patients.

## Supplementary material

10.2196/77390 Multimedia Appendix 1 Qualtrics eligibility screening survey questions.

10.2196/77390 Checklist 1 COREQ checklist.

## Abbreviations

AI: artificial intelligence
COREQ: Consolidated Criteria for Reporting Qualitative Research
EHR: electronic health record
GPT: generative pretrained transformer
HCP: health care professional
LLM: large language model
