# Supplementary material for: Evaluation of Cancer Survivors’ Experience of Using AI-Based Conversational Tools: Qualitative Study
Source: JMIR Cancer. 2025 Nov 14;11:e77390. doi: 10.2196/77390 (PMC12617959; doi:10.2196/77390)
Supplement: Multimedia Appendix 1 [file cancer-v11-e77390-s001.docx]

**Appendix S1. Qualtrics Eligibility Screening Survey Questions**

**Evaluation of AI-based Conversational Tools on Patient-Provider Communication for Cancer Survivors**

1. The NIH-National Cancer Institute defines a "cancer survivor" as anyone diagnosed with cancer, from the moment of diagnosis through the rest of their life. This term encompasses individuals currently living with cancer as well as those who are cancer-free, aiming to represent all those with a history of cancer rather than impose a label that may not resonate with everyone. Based on this definition, are you are cancer survivor?
   1. Yes
   2. No
2. Are you a survivor of breast and/or prostate cancer?
   1. Yes
   2. No
3. Are you currently in treatment for cancer?
   1. Yes
   2. No
4. What is your name?

[short answer]

1. What is your email address?

[short answer]

1. What is your sex?
   1. Male
   2. Female
   3. Prefer not to answer
2. What is your age?
   1. 18 – 34
   2. 35 - 49
   3. 50 – 64
   4. 65 – 79
   5. 80+
3. What is your ethnicity? (Please select all that apply.)
   1. American Indian or Alaska Native
   2. Black or African American
   3. Native Hawaiian or Other Pacific Islander
   4. White
   5. Asian
   6. Middle Eastern or North African
   7. Hispanic or Latino
4. What is your highest level of education?
   1. High school or GED
   2. Associate degree
   3. Bachelor’s degree
   4. Graduate level degree
5. What is your current employment status?
   1. Full time
   2. Part time
   3. Unemployed
6. Are you self-employed?
   1. Yes
   2. No
7. Do you identify as having any condition(s) that affect day-to-day functioning?
   1. Yes
   2. No
8. Are you a native speaker of English?
   1. Yes
   2. No
9. Do you live in a rural area?
   1. Yes
   2. No
10. Are you a current resident of the United States?
    1. Yes
    2. No
11. Did you receive care in the United States for cancer?
    1. Yes
    2. No
12. What is your name? What is your email address? What is your sex? What is your age? What is your ethnicity? (Select all that apply) What is your highest level of education? What is your current employment status? Are you self-employed? Do you identify as having any condition(s) that affect day-to-day functioning? Are you a native speaker of English? What best describes your previous virtual care experience? Do you belong to an underserved population (e.g. low income, racial or ethnic minority, immigrant or refuge, member of the LGBTQ+ community, disabled and/or chronically ill, from a rural area, elderly, unhoused, unemployed, and more)? If so, please list.
    1. Yes

[Text box]

- 1. No

**Appendix S2. Interview Guide**

**Cancer Survivor Chatbot Interview Guide**

**Introduction**

Thank you for your interest in our study. Your participation is voluntary, and you have the right to withdraw your consent at any time without any consequences or questions from the research team. You may skip any questions or end your participation at any time for any reason without disclosing your reasons. Rest assured; you will not be penalized in any way.

As mentioned in the consent forms, please note that our sessions will be audio-recorded. All audio recordings will remain anonymous and be stored on a secure, password-protected server at the University of North Carolina (UNC). Only the researchers involved in this study will have access to these recordings for research purposes.

**Instructions**

You will be asked a short series of questions about your experiences and perceptions regarding chatbots and their applications in healthcare. Please feel free to respond at your own pace. There are no time restrictions, so answer naturally and candidly.

If you are ready, we will begin recording now. *(Begin recording once the participant accepts.)*

**Questions**

***Acceptability and Comfort Level***

1. How comfortable are you using an AI chatbot to find answers to your health-related questions?

2. What concerns, if any, do you have about AI chatbots as tools for providing healthcare advice to patients?

3. How did you learn to use AI chatbots?

4. How could your experience using AI chatbots be improved?

***Trust and Adherence***

5. How likely are you to believe and adhere to a response from an AI chatbot?

6. After receiving a response from an AI chatbot, would you follow up with a human medical provider to obtain their professional opinion?

***Concerns, Barriers, and Suggestions***

7. Are there any barriers that prevent you from using AI chatbots for healthcare or cancer-related questions?

8. What suggestions do you have to improve the use of chatbots for patients?
