# Supplementary material for: Evaluation of Cancer Survivors’ Experience of Using AI-Based Conversational Tools: Qualitative Study
Source: JMIR Cancer. 2025 Nov 14;11:e77390. doi: 10.2196/77390 (PMC12617959; doi:10.2196/77390)
Supplement: Checklist 1 [file cancer-v11-e77390-s002.docx]

**Appendix A. COREQ Checklist**

**COREQ Checklist Table for Submission**

| **Domain** | **Item** | **Guide Question / Description** | **Response in Manuscript** | **Page #** |
| --- | --- | --- | --- | --- |
| **Domain 1: Research team & reflexivity** |  |  |  |  |
| **Personal characteristics** |  |  |  |  |
| 1 | Interviewer/facilitator | Which author(s) conducted the interviews? | Interviews conducted by HM, SM, MC | p. 8 |
| 2 | Credentials | What were the researcher’s credentials? | SK (PhD, MPH), HM (MPS), SM (MD), MC (MS) | N/A |
| 3 | Occupation | Occupation of researchers at time of study | All researchers are affiliated with UNC (School of Nursing / CHIP) | N/A |
| 4 | Gender | Gender of interviewers | Not explicitly stated | N/A |
| 5 | Experience & training | Interviewer experience/training | HM, SM trained by MC; shadowed initial interviews | p. 8 |
| **Relationship with participants** |  |  |  |  |
| 6 | Relationship established | Was a relationship established prior to study commencement? | No prior relationship with participants | p. 7 |
| 7 | Participant knowledge of interviewer | What did participants know about researchers? | Purpose of study explained during consent process | p. 8 |
| 8 | Interviewer characteristics | Bias, assumptions, personal interests disclosed? | Not applicable — no disclosure in manuscript | **N/A** |
| **Domain 2: Study design** |  |  |  |  |
| **Theoretical framework** |  |  |  |  |
| 9 | Methodological orientation & theory | Underpinning qualitative approach | Descriptive phenomenological design; thematic analysis used | p. 6, 9 |
| **Participant selection** |  |  |  |  |
| 10 | Sampling | How were participants selected? | Purposive sampling via cancer support orgs | p. 7 |
| 11 | Method of approach | How were participants approached? | Flyers, Qualtrics survey, email invitations | p. 7 |
| 12 | Sample size | Number of participants | n = 21 | p. 10 |
| 13 | Non-participation | Dropouts/refusals | None refused or dropped | p. 7 |
| **Setting** |  |  |  |  |
| 14 | Setting of data collection | Where was data collected? | Virtual via Zoom | p. 8 |
| 15 | Presence of non-participants | Anyone else present during interviews? | No, only participant + researcher | p. 8 |
| 16 | Description of sample | Key participant demographics | Table 1, breast/prostate survivors | pp. 10–11 |
| **Data collection** |  |  |  |  |
| 17 | Interview guide | Questions, prompts, guides provided? | Yes, semi-structured guide in Appendix C | Appendix C, p. 55 |
| 18 | Repeat interviews | Were repeat interviews conducted? | No | p. 8 |
| 19 | Audio/visual recording | Were interviews recorded? | Yes, audio recorded via Zoom | p. 8 |
| 20 | Field notes | Were field notes made? | Yes, during interviews | p. 8 |
| 21 | Duration | Interview duration | 20–30 minutes | p. 8 |
| 22 | Data saturation | Was data saturation discussed? | Yes, explicitly described | p. 9 |
| 23 | Transcripts returned | Were transcripts returned to participants for checking? | No, transcripts not returned | p. 8 |
| **Domain 3: Analysis & findings** |  |  |  |  |
| **Data analysis** |  |  |  |  |
| 24 | Number of data coders | Number of coders involved | Two coders per transcript | p. 9 |
| 25 | Description of coding tree | Was coding tree described? | Yes, parent + subcodes explained | p. 9 |
| 26 | Derivation of themes | Themes identified in advance or derived? | Inductive thematic analysis | p. 9 |
| 27 | Software | Software used for coding | Dedoose v9.2.22 | p. 9 |
| 28 | Participant checking | Did participants provide feedback on findings? | No, participants not involved in analysis | **N/A** |
| **Reporting** |  |  |  |  |
| 29 | Quotations presented | Were participant quotes used to illustrate themes? | Yes, in Tables 2–4 | pp. 12–19 |
| 30 | Data and findings consistent | Consistency between data and findings | Yes, aligned | Throughout Results, pp. 11–19 |
| 31 | Clarity of major themes | Are major themes clearly presented? | Yes, three main themes defined | pp. 11–12 |
| 32 | Clarity of minor themes | Minor themes or divergent cases described? | Yes, discussed within each major theme | pp. 13–19 |
